# Supplementary material for: Human iPSC- and Primary-Retinal Pigment Epithelial Cells for Modeling Age-Related Macular Degeneration
Source: Antioxidants (Basel). 2022 Mar 22;11(4):605. doi: 10.3390/antiox11040605 (PMC9025527; doi:10.3390/antiox11040605)
Supplement: Supplementary file 1 [file antioxidants-11-00605-s001.zip › antioxidants-1641328-supplementary/Supplementary Figure S1.pdf]

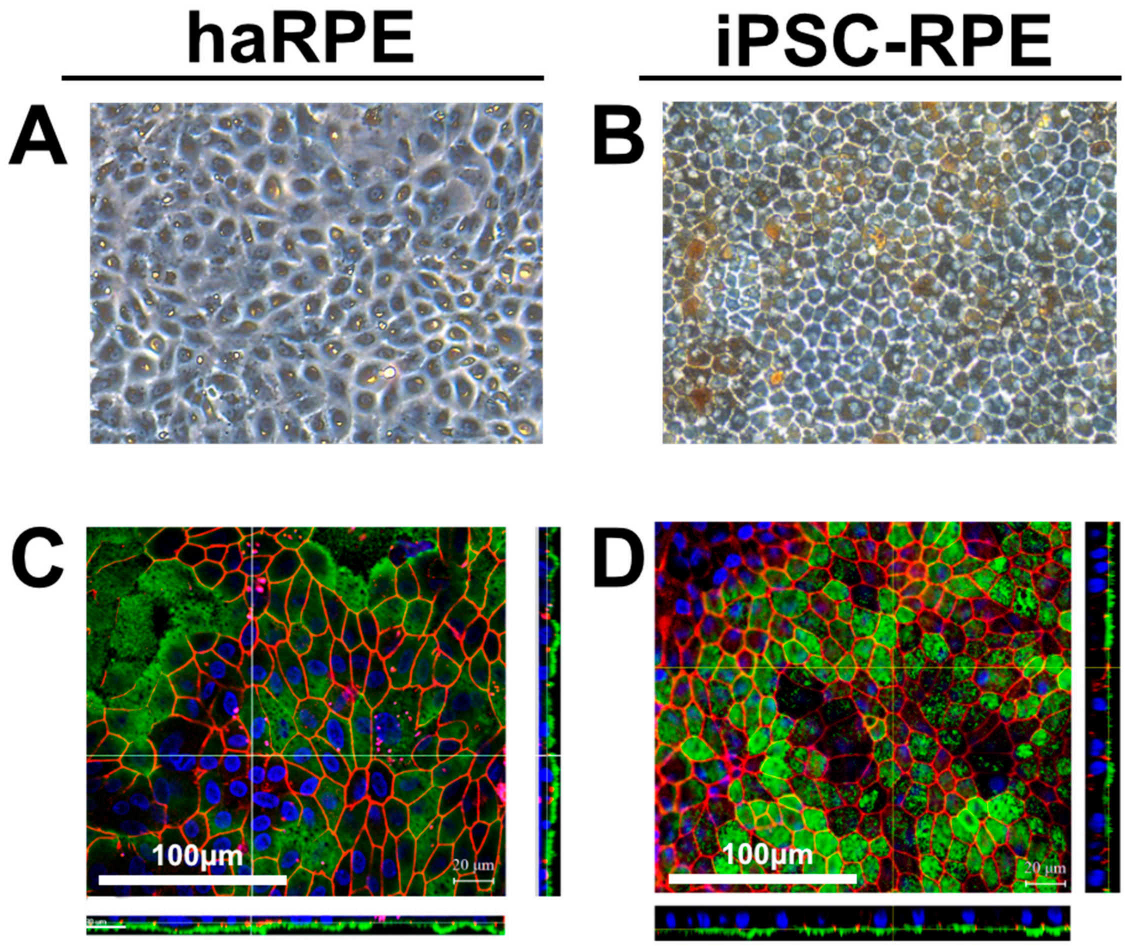

Supplementary Figure S1. Characterization of haRPE and iPSC-RPE cultures. (A,B) Brightfield images of haRPE (A) and iPSC-RPE (B) showing pigment and cobblestone morphology. (C,D) Max intensity Z-projection of 60x images. Immunofluorescent labeling of apical ezrin (green) and tight junction protein ZO-1 (red) in haRPE (C) and iPSC-RPE (D). Nuclei are stained with DAPI (blue). Orthogonal views are shown below and to the right.
