# Supplementary material for: Human iPSC- and Primary-Retinal Pigment Epithelial Cells for Modeling Age-Related Macular Degeneration
Source: Antioxidants (Basel). 2022 Mar 22;11(4):605. doi: 10.3390/antiox11040605 (PMC9025527; doi:10.3390/antiox11040605)
Supplement: Supplementary file 1 [file antioxidants-11-00605-s001.zip › antioxidants-1641328-supplementary/Supplementary Figure S2.pdf]

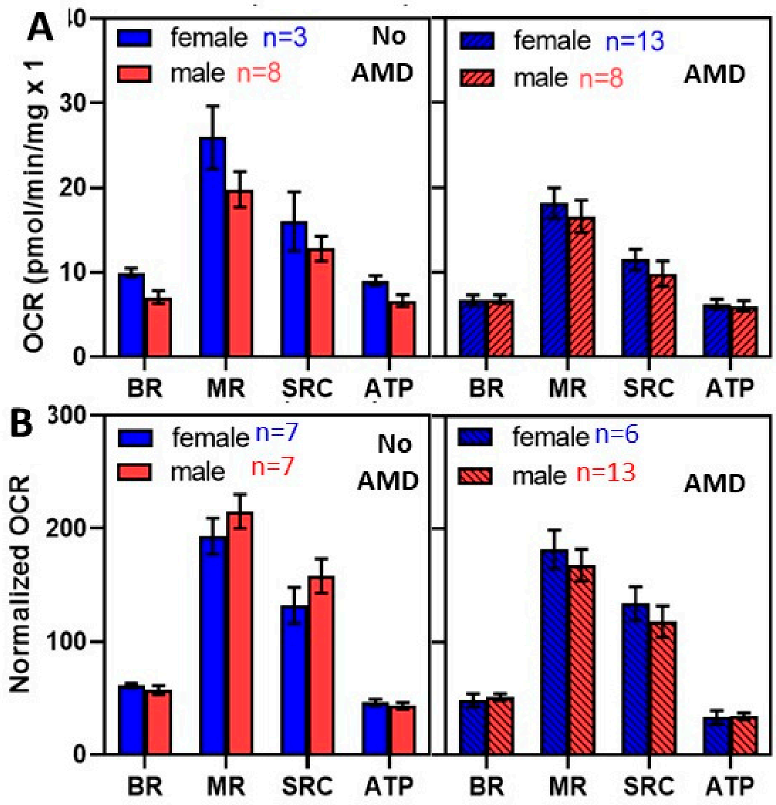

Supplementary Figure S2. Gender Comparison of Mitochondrial Function in haRPE. (A, B) Mitochondrial functional parameters calculated from OCR values of iPSC-RPE (A) and haRPE (B) for females (blue) and males (red). Basal respiration (BR), maximal respiration (MR), spare-respiratory capacity (SRC), and ATP production (ATP). Student's t-test found no significant differences based on gender.
