# Supplementary material for: Human iPSC- and Primary-Retinal Pigment Epithelial Cells for Modeling Age-Related Macular Degeneration
Source: Antioxidants (Basel). 2022 Mar 22;11(4):605. doi: 10.3390/antiox11040605 (PMC9025527; doi:10.3390/antiox11040605)
Supplement: Supplementary file 1 [file antioxidants-11-00605-s001.zip › antioxidants-1641328-supplementary/Supplementary Figure S4.pdf]

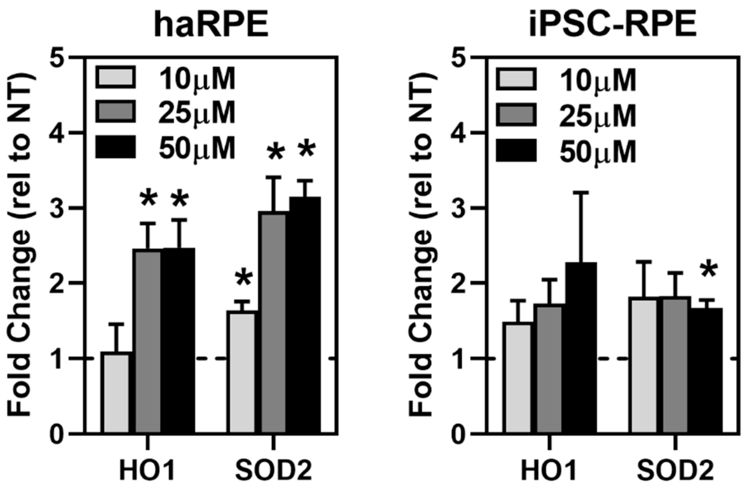

Supplementary Figure S4. Oxidative Stress Response Gene Expression. Gene expression of HO-1 and SOD2 after exposure to 10 μM, 25μM, or 50μM menadione for 24 hours in haRPE (left, n=3) or iPSC-RPE (right, n=3). * denotes $p<0.05$.
