# Supplementary material for: Human iPSC- and Primary-Retinal Pigment Epithelial Cells for Modeling Age-Related Macular Degeneration
Source: Antioxidants (Basel). 2022 Mar 22;11(4):605. doi: 10.3390/antiox11040605 (PMC9025527; doi:10.3390/antiox11040605)
Supplement: Supplementary file 1 [file antioxidants-11-00605-s001.zip › antioxidants-1641328-supplementary/Supplementary Table S1.pdf]

Supplementary Table S1. Donor demographics [A]

Disease State [B]	Sample [C] (n)	Sex Male (n)	Sex Female (n)	Age (Mean ± SD)	Cause of Death [D] (n)
**No AMD haRPE**	12	9	3	71 ± 7.5	Cardiogenic shock (2), CVA (1), multi-system failure (2), Cancer (3), Respiratory Failure (2), Sepsis (2)
**AMD haRPE**	16	7	9	75 ± 8.3	ACE (2), Cancer (1), CVA (1), Heart failure (2), ICH (4), Pneumonia (2), Respiratory Failure (3), Sepsis (1)
**No AMD iPSC-RPE**	7	4	3	70 ± 11.4	ABI (1), ALS (1), ICH (1), Cardiogenic shock (1), multi-system failure (1), Cancer (1), Respiratory Failure (1)
**AMD iPSC-RPE**	13	5	8	75 ± 8.6	ACE (1), Cancer (1), Cardiomyopathy (1), CVA (1), ICH (2), pneumonia (1), PE (1), Respiratory Failure (1), Sepsis (4)

ABI = anoxic brain injury; ACE = acute cardiac event; ALS = amyotrophic lateral sclerosis; CVA = cerebrovascular accident (stroke); ICH = Intracerebral hemorrhage; PE = pulmonary embolism.

[A] Information supplied by Lions Gift of Sight, St. Paul, MN.

[B] Minnesota Grading System (MGS) was used to evaluate the stage of AMD in eye bank eyes (Olsen and Feng, 2004). No AMD = MGS1; AMD = MGS2 and MGS3.

[C] Sample number indicates the total donors with or without AMD used in the current study.

[D] The number of donors for each cause of death is indicated in parentheses.
