# Supplementary material for: Human iPSC- and Primary-Retinal Pigment Epithelial Cells for Modeling Age-Related Macular Degeneration
Source: Antioxidants (Basel). 2022 Mar 22;11(4):605. doi: 10.3390/antiox11040605 (PMC9025527; doi:10.3390/antiox11040605)
Supplement: Supplementary file 1 [file antioxidants-11-00605-s001.zip › antioxidants-1641328-supplementary/Supplementary Table S2.pdf]

Supplementary Table S2. Primer for qPCR analysis

Gene	Gene ID		Sequence
HO-1	3162	Forward	CCAGCGGGCCACAACAAAGT
		Reverse	GCCTTCAGTGCCCACGGTAAGG
SOD2	6647	Forward	GTGTGGGAGCACGCTTACTA
		Reverse	AGAGCTTAACATACTCAGCATAACG
ARBP	6175	Forward	CGACCTGGAAGTCCAACTAC
		Reverse	ATCTGCTGCATCTGCTTG
HPRT1	3251	Forward	TGCAGACTTTGCTTTCCTTGGTCAGG
		Reverse	CCAACACTTCGTGGGGTCCTTTTCA
