# Supplementary material for: Human iPSC- and Primary-Retinal Pigment Epithelial Cells for Modeling Age-Related Macular Degeneration
Source: Antioxidants (Basel). 2022 Mar 22;11(4):605. doi: 10.3390/antiox11040605 (PMC9025527; doi:10.3390/antiox11040605)
Supplement: Supplementary file 1 [file antioxidants-11-00605-s001.zip › antioxidants-1641328-supplementary/Supplementary Table S3.pdf]

Supplementary Table S3. Antibodies used in this study

Antigen	Company	Product
Total OXPHOS Human WB antibody cocktail	abcam	ab110411
pAMPK (Thr172)	Cell Signaling	2535
AMPK	Cell Signaling	2532
VDAC	Cell Signaling	4866
COX IV	Cell Signaling	11967
LC3	Cell Signaling	4108
p62	Cell Signaling	5114
LAMP1	Cell Signaling	9091
Cathepsin D	Cell SIgnaling	2284
