# Supplementary material for: Human iPSC- and Primary-Retinal Pigment Epithelial Cells for Modeling Age-Related Macular Degeneration
Source: Antioxidants (Basel). 2022 Mar 22;11(4):605. doi: 10.3390/antiox11040605 (PMC9025527; doi:10.3390/antiox11040605)
Supplement: Supplementary file 1 [file Supplementary Figure S3.pdf › antioxidants-1641328-supplementary/Supplementary Figure S3.pdf]

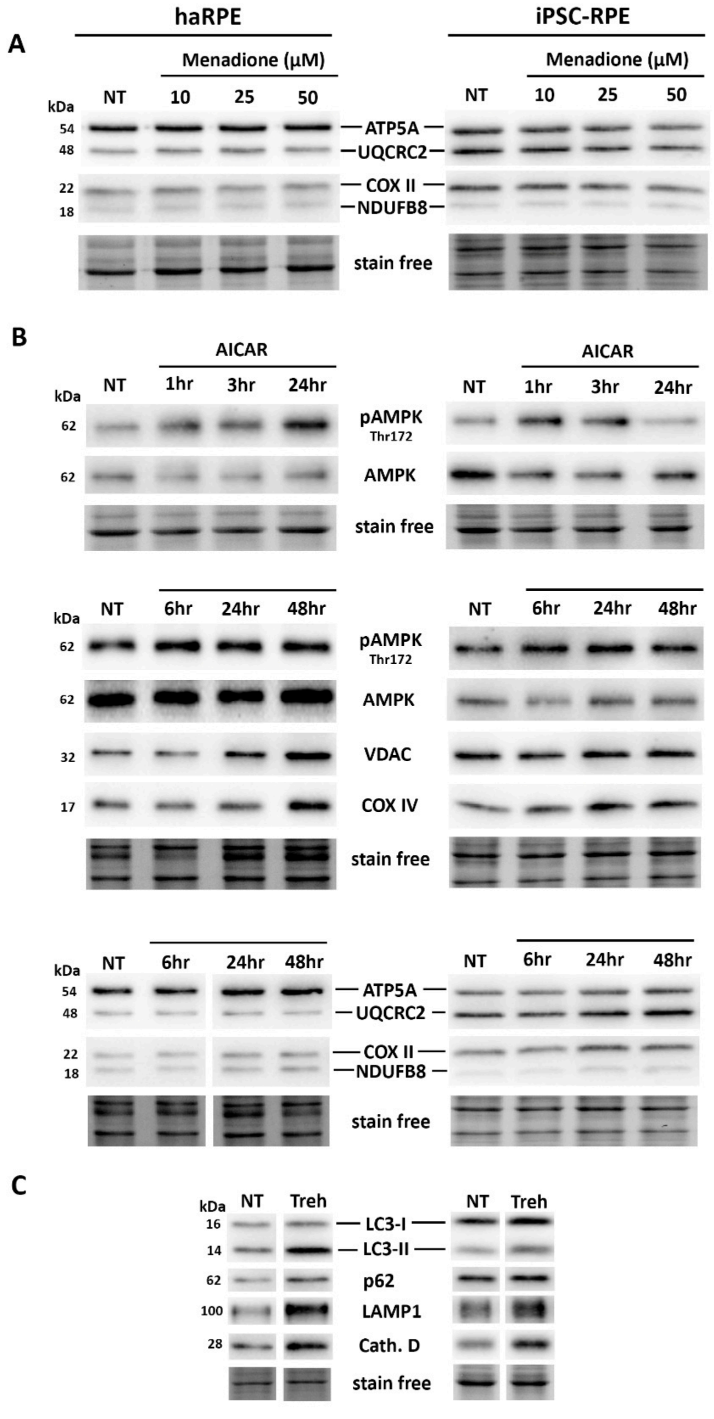

Supplementary Figure S3. Representative immunblots from menadione treatment (A, Fig. 2), AICAR treatment (B, Fig. 3), or trehalose treatment (C, Fig. 4).
